# Associations between lower limb eccentric muscle capability and change of direction speed in basketball and tennis players

**DOI:** 10.7717/peerj.13439

**Published:** 2022-05-23

**Authors:** Darjan Smajla, Žiga Kozinc, Nejc Šarabon

**Affiliations:** 1 University of Primorska, Faculty of Health Sciences, Izola, Slovenia; 2 Innorenew CoE, Izola, Slovenia; 3 S2P, Science to Practice, Ltd., Laboratory for Motor Control and Motor Behavior, Ljubljana, Slovenia

**Keywords:** Deceleration, Braking, Strength, Power, Agility

## Abstract

**Background:**

The ability to perform a quick and rapid change of direction (CoD) is an important determinant of success in a variety of sports. Previous studies have already highlighted that eccentric strength is a dominant predictor of CoD. However, these studies evaluated eccentric strength through a limited number of outcome measures and used small sample sizes.

**Methods:**

A total of 196 athletes participated in the study**.** The aim of our study was to investigate: (1) the correlation between eccentric outcome measures derived from different tests (Nordic hamstring exercise (NHE), countermovement jump (CMJ) and flywheel (FW) squats), (2) the association between eccentric outcome measures and CoD 90°, CoD 180°; and (3) proportion of explained variance in CoD performance.

**Results:**

Very large associations (*r* = 0.783, *p* < 0.001) were observed between peak torque during NHE (NHE_PT_) and force impulse during the eccentric phase of CMJ (CMJ_FI_). Small to moderate correlations were calculated between peak eccentric force in flywheel squats and peak eccentric force in CMJ (*r* = 0.220–035, *p* < 0002). All eccentric CMJ outcome measures and NHE_PT_ were reported as moderate negative associations with both CoD tests. Eccentric measures explained 25.1% of the variance in CoD 90° (CMJ_PF_, NHE_PT_, F_0.125_ –peak eccentric force during FW squats with 0.125 kg m^2^ load), while the same outcome measures explained 37.4% of the variance for CoD 180°.

**Conclusion:**

Our results suggest that different measures of eccentric strength specifically contribute to CoD performance. Therefore, for successful CoD performance, different aspects of eccentric strength training should be considered in testing and training (maximal eccentric strength, eccentric-concentric actions with fast execution).

## Introduction

The ability to perform rapid changes of direction (CoD) is one of important determinants for successful performance in various sports where cutting maneuvers are frequently performed (Gamble et al., 2017). For example, the intensity of the basketball matches increased in the last years, which increased the relevance of CoD in a basketball matches for successful performance (Sugiyama et al., 2021). In soccer, players perform up to 800 cutting movements one game (Taylor et al., 2017), while tennis players perform a great number of quick and rapid changes in lateral direction (Kovacs, 2009). The execution of CoD actions requires different types of muscle contraction during braking (eccentric), plant phase (isometric) and propulsion (concentric) for successful deceleration and reacceleration (Spiteri et al., 2014). Consequently, CoD ability is dependent on multiple types of strength (Nimphius, McGuigan & Newton, 2010; Castillo-Rodriquez et al., 2012).

Until now, studies have investigated associations between CoD performance and different physical ability parameters, such as maximal lower body isometric (Spiteri et al., 2014) and dynamic strength (Nimphius, McGuigan & Newton, 2010; Spiteri et al., 2014), lower limb power output during different tasks (Young, James & Montgomery, 2002; Hori et al., 2008), reactive strength during drop jump (Meylan et al., 2009), ground reaction forces during counter movement jump (CMJ) (Spiteri et al., 2014) and more. Several studies underscored the importance of isometric, concentric and eccentric strength for CoD performance. High associations (*r* = −0.79 to −0.89) were found between CoD performance (*T*-test, 505 test) and performance during different strength and power tests (mid-thigh pull, squat, vertical jumps) in female basketball players (Spiteri et al., 2014). In males, lower associations (*r* = −0.57 to −0.62) were found between isometric mid-thigh pull (peak force and force impulse) and 505 test, and between eccentric knee flexion and extension strength with 180° turn CoD (Jones et al., 2017). Moreover, significant moderate associations were found between CoD performance and 1-repetition maximum during hang power clean, front squat, and peak power and height during counter-movement jump (*r* = −0.38 to −0.51) (Hori et al., 2008). However, one of the studies reported that concentric-based tests appear to be poor predictors of CoD performance (Chaabene, 2017).

One of the recent studies showed that in most team sports there is a greater frequency of high and intensive decelerations compared to accelerations (Harper, Carling & Kiely, 2019). While eccentric strength possibly plays the most important role in deceleration, most of the previous studies investigated associations of concentric and isometric outcome measures with CoD performance. There is a paucity of research investigating the importance of eccentric muscle strength for CoD performance. A study on a small number (*n* = 12) female basketball players suggested that eccentric strength (greater vertical braking in eccentric squat) is a deterministic factor and dominant predictor of CoD performance, compared to concentric (concentric squat) and isometric (midthigh pull) strength (Spiteri et al., 2014). Moreover, Greig & Naylor (2017) showed that isokinetic eccentric knee strength measures are better predictors of CoD performance than corresponding concentric measures in rugby and soccer players (*n* = 19). Moreover, Jones, Bampouras & Marrin (2009) emphasized eccentric muscle knee flexor strength as an important determinant for CoD performance in university students (*n* = 38). Furthermore, Jones et al. (2017) showed that stronger female soccer players (*n* = 18) have faster approach velocity and a greater reduction in velocity during the contact and consequently a better braking ability (eccentric strength) and faster overall performance during 180° CoD test. The importance of eccentric muscle ability for CoD performance is also supported by evidence from intervention studies using eccentric training. For example, training content such as squatting on flywheel (FW) device significantly improved CoD performance (De Hoyo et al., 2016; Tous-Fajardo et al., 2016) and compared to traditional resistance exercise using free weights (Coratella et al., 2019).

All the previous studies which investigated the associations between different outcome parameters and CoD performance were performed on a small number of participants (up to 38). It remains unknown whether different eccentric strength outcome measures (*e.g.*, obtained with single or multi-joint test, bodyweight or loaded tests, *etc.*) exhibit similar or different association with CoD performance. It could also be that the associations add up, meaning that a combination of the multiple outcome measures explain a larger proportion of the variance than a single outcome measure. Therefore, our first aim was to investigate the associations between different outcome measures of eccentric muscle ability during different tasks (Nordic hamstring exercise (NHE), squatting on flywheel (FW) device and CMJ). We hypothesized that associations between eccentric outcome measures during different tasks will be small to moderate as each task represents a specific type of eccentric muscle ability (Jones, Bampouras & Marrin, 2009; Spiteri et al., 2014). Our second aim was to investigate the associations between different outcome measures of eccentric muscle ability and CoD performance (90° and 180° turn). We hypothesized that outcome measures of eccentric muscle ability will be in a significant negative correlation with CoD performance, while higher negative associations will be calculated for CoD 180° time (Harper, Jordan & Kiely, 2021). Moreover, we anticipated higher associations will be calculated between CoD test and eccentric outcome measures during complex movements (CMJ, FW squats), compared to eccentric outcome measures form the local strength test (Nordic hamstring exercise (NHE), in light of the high similarity between CoD, CMJ and FW tasks (all being multi-joint movements, primarily executed by lower limbs). The third aim of our study was to investigate the portion of explained variance in CoD performance with the observed eccentric outcome measures. We hypothesized that our selected eccentric outcome measures will explain at least 30% of the variance in CoD 180° time and at least 20% of the variance in CoD 90° time based on previous literature which suggests larger ground contact times during cuts with higher angle (Dos’Santos et al., 2018).

## Materials & Methods

### Participants

A total of 196 categorized athletes from basketball and tennis participated in the study. Specific information about our participants is provided in Table 1. All participants were recruited through national sports associations. Participants with any lower leg injuries in the past 6 months or neurological disorders were excluded from the study. All participants or their parents (legal guardians) signed informed consent after the experimental procedure had been presented to them. The experiment was approved by the Republic of Slovenia National Medical Ethics Committee (approval no. 0120-690/2017/8) and was conducted in accordance with the latest revision of the Declaration of Helsinki.

**Table 1:** Basic participant data.

Sport		N	Age (years)	Body height (cm)	Body mass (kg)	Training years	Weekly training sessions
Basketball	Female Male All	41 66 107	16.7 ± 1.5 16.7 ± 1.1 16.7 ± 1.3	174.6 ± 5.6 189.3 ± 8.0 183.6 ± 1.5	70.6 ± 12.0 81.0 ± 12.7 77.1 ± 13.4	5.4 ± 1.3 6.0 ± 1.9 5.8 ± 1.7	9.7 ± 1.8 9.6 ± 2.5 9.6 ± 2.2
Tennis	Female Male All	36 53 89	16.5 ± 2.6 16.5 ± 3.4 16.5 ± 3.1	170.1 ± 5.6 178.1 ± 8.0 174.8 ± 8.1	62.2 ± 7.3 68.3 ± 11.1 65.9 ± 10.1	6.8 ± 3.5 6.4 ± 3.1 6.6 ± 3.3	8.2 ± 2.4 7.3 ± 3.1 7.7 ± 2.9
All		196	16.6 ± 2.3	179.6 ± 10.2	71.9 ± 13.2	6.1 ± 2.5	8.7 ± 2.7

**Notes.**

N number of participants

### Sample size calculation

Although cross-sectional studies in sport sciences that utilize correlational analysis usually use smaller sample sizes (usually on the order of 10), this is statistically inappropriate. For a small effect size (*d* = 0.4) to reach 80% statistical power, at least 50 participants are needed even in the simplest research design (Brysbaert, 2019). Moreover, strong arguments have been made that estimating the effect size from smaller pilot studies or previous studies can lead to vastly erroneous estimations (Brysbaert, 2019). Instead, the smallest worthwhile effect should be considered in sample size calculations. For the correlational analysis, 194 participants appear to be needed to reach 80% of statistical power for *r* = 0.2 (corresponding roughly to small but not trivial effect size, *d* = 0.4) (Brysbaert, 2019).

### Study design and procedures

**Figure 1:**
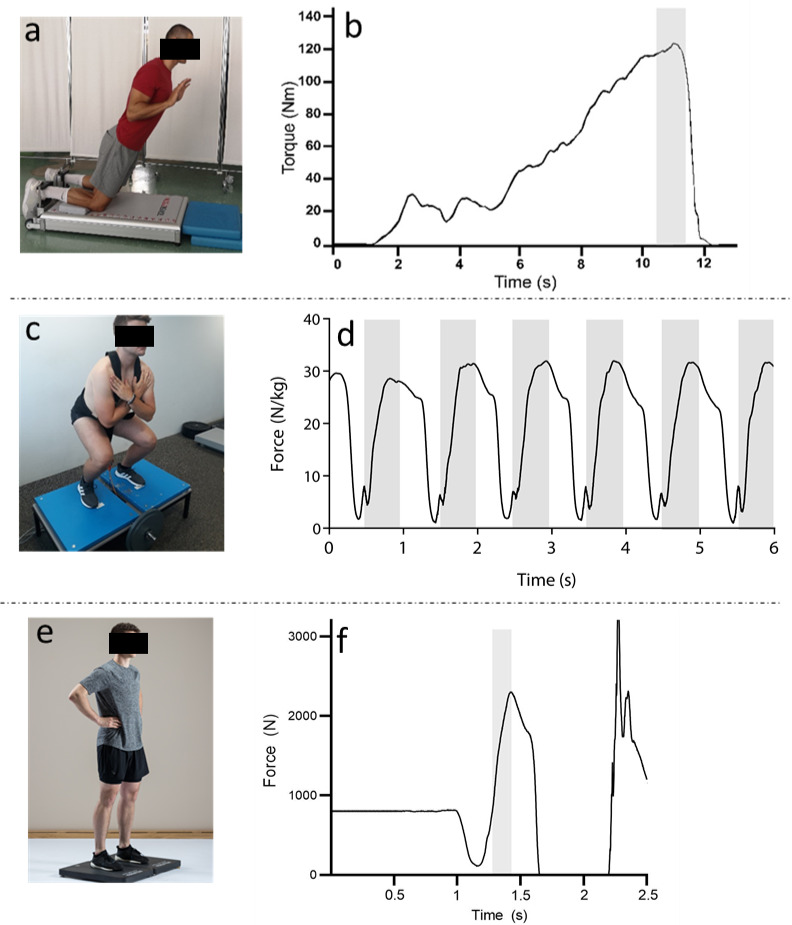
Measurement set up. (A, Nordic hamstring exercise; C, flywheel squats; E, counter movement jump) and sample recordings for each task (B, torque during Nordic hamstring exercise; D relative force during flywheel squats; F force during counter movement jump). Shaded part on sample recordings (B, D and F) represent the portion of the selected task from which outcome parameters were calculated. Braking phase for the CMJ was considered as the best representative of eccentric strength ability.

This was a cross-sectional study, with measurements performed in a single session which lasted approximately 3 h. Before the measurements, participants performed a standardized 20-min warm up which consisted of 10-min of light jogging, 5-min of dynamic stretching exercises and 5-min of low-intensity resistance exercises and activation exercises (heel raises, squat crunches, push-ups, squat jumps and CMJs). All participants performed CoD tasks and three different tests to assess eccentric muscle ability was evaluated: NHE, squatting on FW device and CMJ (Fig. 1). The order of the tests was randomized. Loud verbal encouragement was provided during all tests to ensure maximal effort.

### Change of direction

**Figure 2:**
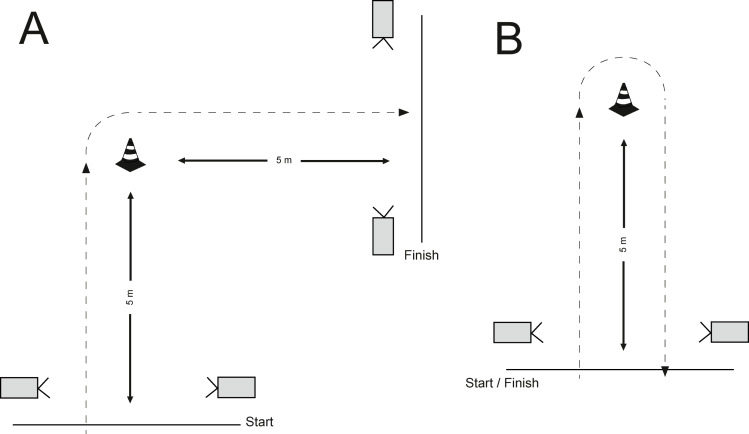
Representation of CoD 90° and CoD 180° tasks.

CoD tests were conducted in a gym with tartan floor. Tests were timed using photocell timing gates (Brower Timing Systems, Draper, UT, USA). Timing gates were placed at hip height, while the starting line was 0.5 m behind the first timing gate (to avoid early triggering). Before the measurements, each participant performed two warm-up trials (for each CoD test: 90° and 180°) at 50 and 75% of their maximal speed. After that each participant performed 3 maximal CoD trials per side turn (left and right, random order) and task (90° and 180°). The rest period between each repetition was 1-min, while there was a 3-min break between the tasks (CoD 90° and CoD 180°) (12 trials in total). For both tests, participants were instructed to place one foot on the middle of the starting line. In 90° CoD participants sprinted from starting position to the cone and made a 90° turn on one of the sides and sprint through the finish line (Fig. 2A, both timing gates used). In 180° CoD the, the participants sprinted around the cone and back to the first timing gate (Fig. 2B, one timing gate used). In both test the total distance was 10 m. All participants were instructed to start at their own will. For each task (CoD 90°, CoD 180°) time (s) was calculated as an average of best repetitions for the left and right side (CoD 90° = (best CoD 90° left + best CoD 90° right)/2; CoD 180° = (best CoD 180° left + best CoD 190° right)/2).

### Nordic hamstring exercise

The assessment of knee flexor strength was performed using a valid and reliable NHE device (Marušič, Marković & Šarabon, 2021). Participants were in a kneeling position. Lower legs were placed on the padded mat distally to the patella to allow the movement of the patella during the NHE descent, while posterior side of the lower leg was in contact with superior part of the sensor brace (approximately five cm proximal to the medial malleoli). After 3 repetitions with submaximal efforts, the participants performed 3 maximal repetitions of bilateral NHE. Participants were instructed to gradually lean forward with the trunk, while maintaining the slowest possible speed and maximally resisting this movement with both lower limbs. The trunk and the hips were in a neutral position during descent, while the hands were held across the chest (Marušič, Marković & Šarabon, 2021). The highest torque (Nm) during the NHE exercise (from all 3 repetition) was considered (NHE_PT_).

### Flywheel squats

The participants performed squats on a custom-made FW device (Smajla, Spudić & Šarabon, 2021). The different equidistant loads were used for each participant: 0.05, 0.125 and 0.2 kg m^2^. Prior to the measurements, the correct technique of squatting on the FW device was demonstrated and described. The participant performed 3 familiarization sets of five to ten submaximal squats with each load to achieve the correct tempo and the amplitude of the execution. The position-time data (braking phase of the squat) of the participant was provided with a displacement sensor (Draw-wire, Way-Con SX-50, Taufkirchen, Germany, range 1250 mm, linearity ±0.02%) which was attached to the FW device (below the standing surface) and between the legs of participants on the lifting harness. A draw-wire sensor was also used for real-time monitoring of the squat amplitude which was displayed on the screen in front of the participant. The ground reaction force data during squats was acquired with a bilateral force plate system (type 9260AA, Kistler Instrumente AG, Winterthur, Switzerland) with Kistler MARS software (Fig. 1). The Draw-wire sensor and force plate were synchronized in time and acquisition with a USB Data Acquisition System (Type 5695B; Kistler Instrumente AG, Winterthur, Switzerland). Participants performed eight squat repetitions with each load (loads were selected in random order). Between each load there was a 2-min break (Sabido et al., 2020). Participants were instructed to perform the first two repetitions only to accelerate FW and stabilize the amplitude of the squat (these two repetitions were excluded from analysis). The next six repetitions were performed at maximal effort and were used for analysis based on our previous findings (Spudić, Smajla & Šarabon, 2020). Squats were performed from the 90° knee angle to full extension (0° knee angle), while ankle extension was not allowed. Meanwhile, arms were crossed across the chest. Participants performed the concentric phase of the squat as quickly as possible, delaying the braking during the first third of the eccentric phase to make the transition from the eccentric to concentric phase of the squat as short as possible. Peak eccentric force (N) was calculated as an average of the six consecutive repetitions for each load (*F*_0.05_, *F*_0.125_, *F*_0.2_).

### Countermovement jump

The CMJ was performed on a bilateral force plate (model 9260AA6; Kistler, Winterhur, Switzerland). Before the testing, participants performed three familiarization trials at 80% of their estimated maximal effort to ensure proper jump execution. After that, participants performed three maximal bilateral CMJs with 30 s rest between each jump which was already shown as a reliable protocol (Šarabon et al., 2020). They were instructed to put their hands on the hips and maintain their position during the jump and jump as high as possible by performing a fast countermovement (approximately at the knee angle at 90° ) and push-off with hip, knee and ankle extension. Participants’ squat depth was previously determined and controlled with an elastic band at the appropriate height behind the participant. The outcomes for CMJ were derived from the braking phase (McMahon et al., 2018), which was considered as best representative of the eccentric strength ability. The following parameters were calculated using MARS software: force impulse during the braking phase (CMJ_FI_, Ns), peak eccentric force (CMJ_PF_, N/kg) and maximal rate of force development (CMJ_RFD_, Ns).

### Statistical analysis

Statistical analyses were performed in SPSS (version 25.0, SPSS Inc, Chicago, USA). Descriptive statistics are reported as mean ± standard deviation. The normality of the data distribution was tested with Shapiro–Wilk test. The associations were assessed with Pearson correlation coefficients. Qualitative interpretations of the correlation coefficients were interpreted as follows: (0.00–0.19 trivial; 0.20–0.29 small; 0.30–0.49 moderate; 0.50–0.69 large; 0.70–0.89 very large; 0.90–0.99 nearly perfect; 1.00 perfect) (Hopkins et al., 2009). Multiple linear stepwise regressions were done with CoD 90° and CoD 180° as dependent variables, while all eccentric outcome measures (NHE_PT_, *F*_0.05_, *F*_0.125_, *F*_0.2_, CMJ_FI_, CMJ_PF_, CMJ_RFD_) were included as candidate predictors. Collinearity diagnostic tests were performed. We conservatively set the thresholds for the presence of collinearity at ≤0.3 for tolerance and ≥3 for variance inflation factor (Belsley, Kuh & Welsch, 1980). The threshold for statistical significance was set at *α* < 0.05.

## Results

### Associations between eccentric outcome measures

**Figure 3:**
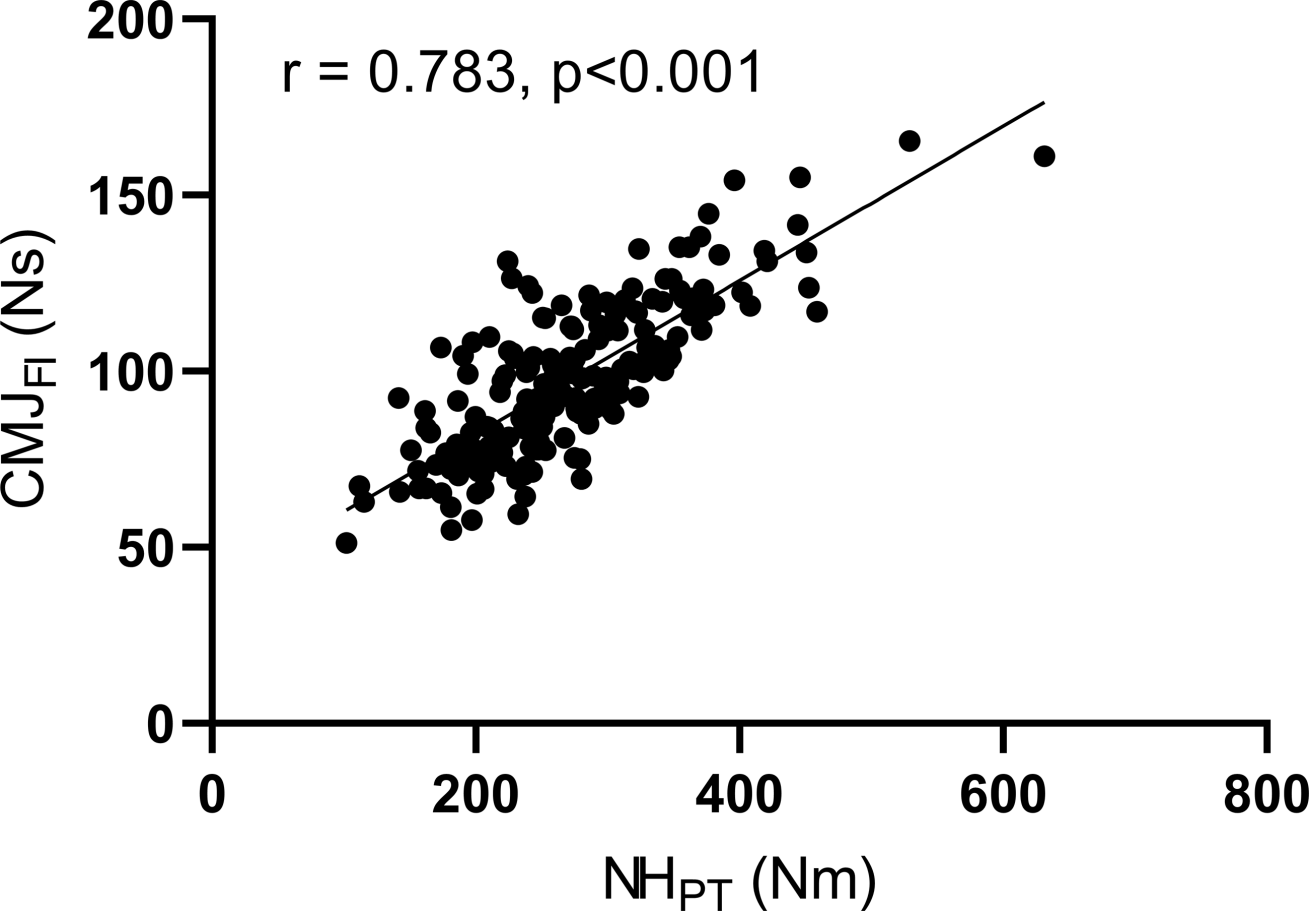
Pearson correlations between force impulse during eccentric part of CMJ (CMJ_FI_) and the peak torque during Nordic hamstring exercise (NH_PT_).

Very large and statistically significant correlations were present between NHE_PT_ and CMJ_FI_ (*r* = 0.783, *p* < 0.001) (Fig. 3), while significant moderate correlations were calculated between NHE_PT_ and CMJ_RFD_ (*r* = 0.379, *p* < 0.001), as well as between *F*_0.05_ and CMJ_PF_ (*r* = 0.335, *p* < 0.001). *F*_0.05_ and *F*_0.0125_ were in small significant correlation with CMJ_PF_ (*r* = 0.22−0.224, both *p* = 0.002).

### Associations between eccentric outcome measures and CoD time

**Figure 4:**
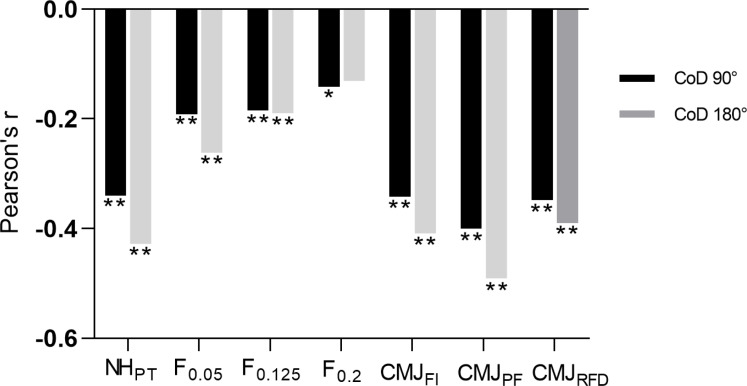
Pearson correlations between eccentric outcome measures. (NH_PT_ –Nordic hamstring peak torque, F_0.05_ –peak eccentric force on flywheel (FW) device with 0.05 kg m^2^ load, F_0.125_ –peak eccentric force on FW device with 0.125 kg m^2^, F_0.2_ –peak eccentric force on FW device with 0.2 kg m^2^, CMJ_FI_ –force impulse during eccentric phase of counter movement jump (CMJ), CMJ_PF_ –peak eccentric force during CMJ, CMJ_RFD_ –maximal eccentric rate of force development during CMJ) and change of direction (CoD) time at 90° (CoD 90° and 180° turn (CoD 180°). An asterisk (*) indicates *p* < 0.05; two asterisks (**) indicate *p* < 0.001.

The CoD 90° time and CoD 180° time were in strong correlation (*r* = 0.77; *p* < 0.001). The highest negative correlations were seen between CoD 90° time and all outcome measures of CMJ and NHE_PT_ (*r* = from −0.342 to −0.400, moderate correlations, *p* < 0.001), while CoD 90° time was in trivial negative correlation with FW outcome measures (*r* = from −0.14 to −0.19, *p* = 0.008–0.049) (Fig. 4).

CMJ outcome measures and NHE_PT_ were in moderate negative significant correlation (*r* = from −0.390 to −0.491, *p* < 0.001) CoD 180°. Trivial to small correlations between CoD 180° with *F*_0.125_ and *F*_0.05_ (*r* = −0.190–0.262, *p* = 0.001–0.008) were observed (Fig. 4).

### Regression analyses

Collinearity was not detected in our regression analyses (all tolerance values > 0.3, all VIF < 3.0). In regression model for CoD 90° and CoD 180° (dependent variables) we included all calculated parameters (NHE_PT_, *F*_0.05_, *F*_0.125_, *F*_0.2_, CMJ_FI_, CMJ_PF_, CMJ_RFD_).

Following eccentric outcome measures included in our study explained 25.1% of the variance in CoD 90°: CMJ_PF_ (*β* = −0.431, *p* < 0.001), NHE_PT_ (*β* = −0.232, *p* < 0.001), *F*_0.125_ (*β* = −0.138, *p* = 0.034), *p* < 0.001). In stepwise model with one predictor 16% of variance was explained with CMJ_PF_ (*β* = −0.400, *p* < 0.001) , while 23.3% was explained with two predictors: CMJ_PF_ (*β* = −0.352, *p* < 0.001) and NHE_PT_ (*β* = −0.276, *p* < 0.001).

In CoD 180° 37.4% of variance was explained with CMJ_PF_ (*β* = −0.398, *p* < 0.001), NHE_PT_ (*β* = −0.364, *p* < 0.001) and *F*_0.125_ (*β* = −0.132, *p* = 0.027). In stepwise model with one predictor 24.1% of variance was explained with CMJ_PF_ (*β* = −0.491, *p* < 0.001), while 35.8% of variance was explained with two predictors: CMJ_PF_ (*β* = −0.430, *p* < 0.001) and NHE_PT_ (*β* = −0.348, *p* < 0.001).

## Discussion

The purpose of this study was to investigate the associations between eccentric strength outcome measures assessed through different tasks, and their relationship with CoD performance. Very large associations were seen between T_NHE_ and CMJ_FI_, while moderate associations were seen between NHE_PT_ - CMJ_RFD_, and *F*_0.05_ - CMJ_PF_. All eccentric CMJ outcome measures and NHE_PT_ were in moderate negative associations with both CoD tests, while associations of CoD with FW measures were trivial to small. Our eccentric outcome measures explained 25.1% of the variance in CoD 90° (specifically, CMJ_PF_, NHE_PT_, *F*_0.125_), while the same outcome measures explained 37.4% of the variance for CoD 180°.

Previous studies have suggested that eccentric muscle ability plays an important role in CoD performance (Jones, Bampouras & Marrin, 2009; Spiteri et al., 2014; Greig & Naylor, 2017; Jones et al., 2017). Nevertheless, these studies mostly investigated single eccentric outcome measure in relation to CoD performance. In our study, we investigated different eccentric outcome measures from different tasks that reflect various aspects of eccentric muscle ability. In NHE, the maximal eccentric strength of hamstring muscles was assessed, while in FW squats and CMJ, we investigated outcome measures that assessed participants’ ability to brake/decelerate under body mass only and additional load conditions. Our results revealed very large correlations between NHE_PT_ and CMJ_FI_ (Fig. 3) and moderate correlations between NHE_PT_ and CMJ_RFD_. Participants with higher NHE_PT_ were able to brake/decelerate more intensively and produce greater CMJ_FI_ and CMJ_RFD_ during the eccentric phase of CMJ. These associations confirmed the assumption that maximal eccentric strength is an important factor for successful and fast deceleration (Kovacs, Roetert & Ellenbecker, 2008). The peak eccentric force during the squats on the FW device with 0.05 kg m^2^ (*F*_0.05_) load was in moderate correlation with peak eccentric force during CMJ (CMJ_PF_). Participants with greater eccentric peak force during FW squats with the 0.05 kg m^2^ load produced greater peak eccentric forces also during CMJ. In this case, we expected even greater correlations because a similar outcome measure is evaluated in body mass only (CMJ) and additional load (FW) conditions. During CMJ, slow reactive strength is present as the approximate duration of CMJ is 0.5 s. Movement velocities during FW with low FW load mimic patterns observed in CMJ task (Bobbert & Van Ingen Schenau, 1988), while FW exercises with higher loads are performed slower, and their transfer to functional movement tasks might be limited (Spudić et al., 2021). Consequently, these different strength capability requirements yielded smaller correlations between CMJ_PF_ and *F*_0.05_, and CMJ_PF_ and F0_0.125_ Based on our results we can reject our first hypothesis as very large correlations were found between the two outcome measures (NHE_PT_ and CMJ_FI_).

Regarding the correlations between the eccentric outcome measures and CoD in this study, negative moderate correlations were seen between all CMJ outcome measures, NHE_PT_ and CoD 90° time (*r* = from −0.342 to −0.400). Similar, but slightly higher correlations were found between all CMJ outcome measures, NHE_PT_ and CoD 180° time (*r* = from −0.390 to −0.491) where higher deceleration demands are present. Only trivial to small correlations were found between CoD times and peak eccentric force during FW squats. These results can be explained by the fact that during CoD and CMJ, a combination of sudden braking, acceleration and short contact time (stretch-shortening cycle) are needed for a successful task performance (Bosco, Komi & Ito, 1981). In CoD, a participant must exert a great amount of force to the ground in short time (similar to CMJ contact time) (Nygaard Falch, Guldteig Rædergård & Van den Tillaar, 2019), while overcoming only his/her body mass. It seems that FW squats with higher load are less associated with CoD performance. However, one of the intervention studies showed that FW training with higher loads is more beneficial for CoD improvement (De Hoyo et al., 2015). Therefore, the small associations observed in ours study do not necessarily imply that training with higher FW loads should be avoided, as they better promote neural and muscular adaptations during eccentric resistance exercise (English et al., 2014). A moderate negative correlation between CoD time and NHE_PT_ was expected as knee flexors are important contributors to body propulsion. Based on previous studies that showed very large correlations between 505 CoD test (*r* = 0.88) and eccentric back squat strength (Spiteri et al., 2014), we expected even greater associations. However, in our case, we used CoD 90° where the change of direction angle is smaller, while CoD 180° was performed around the cone. Furthermore, eccentric back squat (Spiteri et al., 2014) may better stimulate strength requirements during CoD (upright position) compared to NHE used in our case. Based on our results we can confirm our second hypothesis as all eccentric outcome measures were in a significant negative association with CoD time (except *F*_0.2_ with CoD 180° time), while in general, a slightly higher negative correlations of were seen between eccentric strength capability and CoD 180° time.

One of the most interesting findings of the study was that the percentage of the common variance among CoD performance and eccentric capability is dependent on the CoD angle (*i.e.,* 25.1% of the common variance in CoD 90° time and 37.4% for CoD 180° time). Based on our results we can confirm our third hypothesis. Recent evidence has highlighted several biomechanical differences among CoD tasks with different CoD angles (Dos’Santos, Thomas & Jones, 2021), which may explain our results. For instance, larger peak knee flexion and hip flexion amplitudes, are observed in CoD with CoD larger angles (Dos’Santos, Thomas & Jones, 2021). Moreover, larger ground contact times, but lower approach velocities are seen during cuts with higher angle (Dos’Santos et al., 2018). In CoD 180°, the ability to rapidly reduce the velocity over the penultimate foot contact through the plant step is of paramount importance for faster performance (Jones et al., 2017). Since the ability to decelerate efficiently is underpinned by eccentric strength capacity (Harper, Jordan & Kiely, 2021), it is not surprising that a larger amount of variance in CoD 180° time (compared to CoD 90° time) was explained in our study by eccentric capability parameters. The performances of CoD 90° and CoD 180° were strongly related in the present study (*r* = 0.77); nevertheless, our results, together with previous studies, suggest that different strength determinants are underlying the performance of CoD tasks executed at different angles.

This is the first study in the field of CoD performance which evaluated multiple outcome measures from different specific eccentric tasks with this number of participants. However, there are a few limitations that should be considered. All evaluated tests were performed bilaterally, while CoD tasks comprise unilateral actions. Unilateral tasks may better simulate CoD execution (Meylan et al., 2009) which may consequently yield greater correlations and percentage of the explained variance. Participants were selected from two different sports which may also affect the results. Participants were not experienced in flywheel training; consequently longer familiarization protocol should be performed prior the study. Moreover, some of our participants were early adolescents who may not yet develop their CoD performance, which increases rapidly throughout adolescence (Uzun & Abbulut, 2006). Furthermore, for even better insights in “pure” CoD performance, a calculation of CoD deficit (*i.e.,* subtracting the time to complete equidistant liner sprint from the total CoD task time) should be considered.

## Conclusions

The present study demonstrated moderate to large correlations between different eccentric outcome measures obtained from CMJ, NHE and FW squat. Even though our selected outcome measures are inter-related, the modest magnitudes of the observed associations show that each task represents a specific subtype of eccentric muscle strength and power ability. The highest negative correlations with CoD times were observed for NHE_PT_ and all CMJ outcome measures, while eccentric peak forces during FW squats were in small correlation with CoD performance. At last, eccentric outcome measures explained 25.1% (CoD 90°) and 37.4% (CoD 180°) of variance, whereby most important predictors were CMJ_PF_, NHE_PT_ and *F*_0.125_. Our results suggest that different physical determinants of specific eccentric strength ability are underpinning CoD performance. Therefore, for successful CoD performance, different aspects of eccentric determinants should be considered (maximal eccentric strength, eccentric-concentric actions with fast execution) for testing and training.

## Supplemental Information

10.7717/peerj.13439/supp-1 Supplemental Information 1 Individual dataset.

## Additional Information and Declarations

### Data Availability

The following information was supplied regarding data availability: The raw data is available in the Supplementary File.
